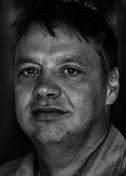# Allosteric Molecular Switches in Metabotropic Glutamate Receptors

**DOI:** 10.1002/cmdc.202000444

**Published:** 2020-08-25

**Authors:** Zoltán Orgován, György G. Ferenczy, György M. Keserű

**Affiliations:** ^1^ Medicinal Chemistry Research Group Research Centre for Natural Sciences Magyar tudósok krt. 2 Budapest 1117 Hungary

**Keywords:** allosteric modulation, GPCRs, metabotropic glutamate receptors, molecular switches, water networks

## Abstract

Metabotropic glutamate receptors (mGlu) are class C G protein‐coupled receptors of eight subtypes that are omnipresently expressed in the central nervous system. mGlus have relevance in several psychiatric and neurological disorders, therefore they raise considerable interest as drug targets. Allosteric modulators of mGlus offer advantages over orthosteric ligands owing to their increased potential to achieve subtype selectivity, and this has prompted discovery programs that have produced a large number of reported allosteric mGlu ligands. However, the optimization of allosteric ligands into drug candidates has proved to be challenging owing to induced‐fit effects, flat or steep structure‐activity relationships and unexpected changes in theirpharmacology. Subtle structural changes identified as molecular switches might modulate the functional activity of allosteric ligands. Here we review these switches discovered in the metabotropic glutamate receptor family..

## Graphical abstract

**Master switch**: Metabotropic glutamate receptors are promising targets for the treatment of several CNS disorders. Their allosteric ligands can modulate the effect of orthosteric ligands, but small changes in the allosteric ligands can cause functional and subtype switching that makes optimizing their medicinal chemistry challenging. Herein, we review the structural background of their modulating effect and suggesting strategies for their optimization.

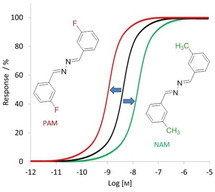

## 1 Introduction

Heterotrimeric G protein‐coupled receptors (GPCRs) constitute the largest class of membrane proteins in the human genome, and are responsible for conveying extracellular to intracellular signals within a broad range of physiological contexts.[^1^, ^2^, ^3^, ^4^] The metabotropic glutamate receptors belong to the class C GPCRs, and they can be classified into eight subtypes divided into three groups based on their pharmacology, sequential homology and G protein coupling. Group I receptors (mGlu_1_, mGlu_5_) are preferentially coupled to G_αq_, and are typically found postsynaptically, Group II receptors (mGlu_2_, mGlu_3_), and Group III receptors (mGlu_4_, mGlu_6_, mGlu_7_ and mGlu_8_) are coupled to G_i/o_, and are found presynaptically except mGlu_6_ receptor which is solely found in the retina.[^4^, ^5^, ^6^, ^7^] Each mGlu monomer contains a so‐called Venus flytrap domain (VFT), a cysteine‐rich domain (CRD) and a seven trans‐membrane domain (7TMD).^[8]^ The first accommodates the extracellular orthosteric binding site. Allosteric binding sites were reported; however, mostly,[^9^, ^10^] but not exclusively,^[11]^ at the 7TMD region of the protein including the one that corresponds to the orthosteric binding site of class A GPCRs.^[12]^ mGlus form mandatory dimers through a disulfide bond at the top of the VFT domains, which are mostly homodimers;[^13^, ^14^] however, heterodimerization was also observed in several cases with other subtypes[^15^, ^16^, ^17^, ^18^] and different GPCRs.[^12^, ^19^, ^20^, ^21^, ^22^]

MGluRs can modulate the release of glutamate and its postsynaptic response, as well as the activity of other synapses.[^23^, ^24^] These receptors have been recognized as therapeutic targets in a number of central nervous system (CNS) diseases, like Parkinson's disease, schizophrenia, epilepsy, ischemia, pain and anxiety.[^23^, ^25^, ^26^] Early attempts to modulate mGlu receptor activity were directed to the orthosteric binding pocket; however, despite some promising attempts in group II[^27^, ^28^] and III receptors,^[29]^ selectively targeting this site proved to be difficult. This can be attributed to the highly conserved nature of the orthosteric binding pocket across the eight receptors. Moreover, as the orthosteric ligands are mostly glutamate derivatives, the bioavailability and blood‐brain barrier permeability of these compounds are usually less than satisfactory.[^5^, ^6^, ^7^]

**Figure 1:**
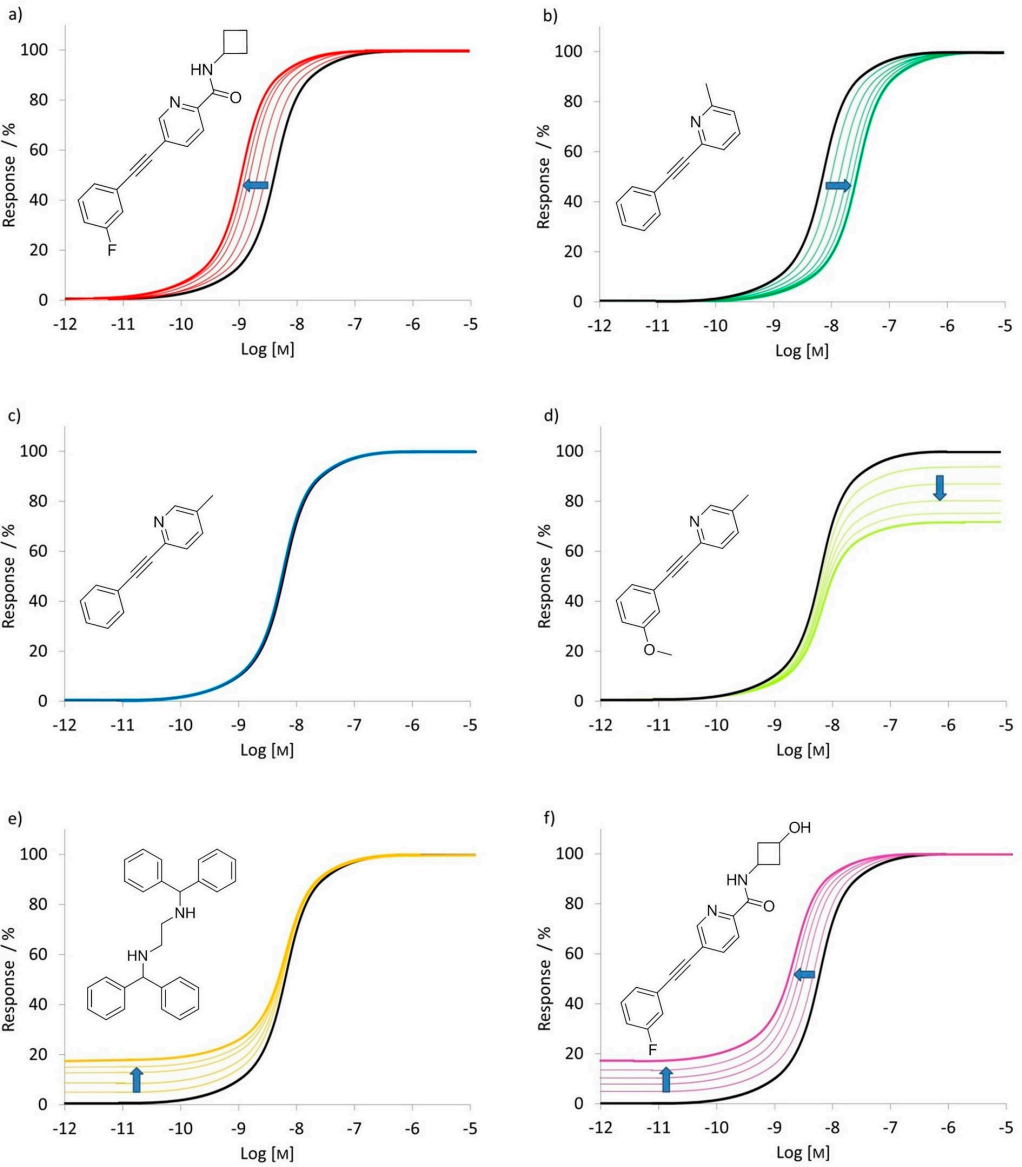
Schematic representation of the effect of allosteric modulators on orthosteric modulation. Dose‐response curves of glutamate are depicted in black, a) PAM, b) NAM, c) NAL, d) PA, e) AA, f) ago‐PAM. Thickened lines represent the maximal shift of dose‐response curves. Examples of the different modes of action within the mGlu family are also provided a) VU0403602, b) MPEP, c) 5MPEP, d) M‐5MPEP, e) AMN082^[37]^, and f) M1.

Later on, allosteric modulation of mGlus emerged as a more viable strategy to achieve receptor subtype selectivity[^13^, ^30^, ^31^, ^32^] owing to the lower sequence similarity of these sites across mGlus. Moreover, pure allosteric modulators are active only in the presence of an orthosteric ligand, and the modulating effect of these modulators is saturable, which reduces the risk of over sensitization. However, allosteric modulators can promote a global change in the receptor conformation, and in this way they can modulate the affinity, potency or efficacy of the orthosteric ligand in negative or positive direction, and they might also inhibit or increase the G protein coupling.[^4^, ^32^, ^33^, ^34^, ^35^] Allosteric modulators are able to exert their modulating effect through different pharmacological modes of action. Negative allosteric modulators (NAMs) weaken and positive allosteric modulators (PAMs) potentiate the effect of the endogenous ligand, while silent allosteric modulators/neutral allosteric ligands (SAMs/NALs) occupy the allosteric binding pocket without detected pharmacological function. Partial antagonists (PAs) are NAMs that fully occupy the allosteric binding site and induce partial reducing effect, while allosteric agonists (AAs) are able to activate the receptor in the absence of orthosteric ligand by binding to an allosteric site and inducing an active conformation of the receptor. Ago‐potentiators (ago‐PAMs) are functioning as both PAMs and AAs[^4^, ^36^] by inducing allosteric agonism, to varying degrees in the absence of orthosteric ligand, however, potentiate the activation of mGlus when glutamate binds (Figure 1).

In the past few years, a large number of mGlu receptor allosteric modulators have been reported, as reviewed in refs. [38–42]; however, despite the significant effort to develop allosteric mGlu ligands, few compounds have reached the clinic to date.^[43]^ This is probably due to the extremely challenging medicinal chemistry optimization, which is further complicated by allosteric coupling and cooperativity connected with variations in functional activity and/or selectivity. In addition, the structure‐activity relationship (SAR) is often either flat or steep, and its transferability between chemotypes is limited.[^43^, ^44^, ^45^] Moreover, the molecular switches that cause variations in affinity versus efficacy modulation, can further complicate the design and optimization.^[43]^

Except for one mGlu_7_ receptor allosteric modulator,^[46]^ allosteric binding pockets of mGlus are located in the 7TMD region of the proteins, Although, our structural knowledge on this region is still limited especially for Group II and III mGlus, subtype selectivity can be achieved due to the unique residues and, hence, the different shape of the 7TMD pockets of mGlus.^[47]^ However, in line with similar observations in several other GPCRs,[^48^, ^49^, ^50^] the 7TMD of mGlu5 was proposed to be a functional water channel.[^51^, ^52^] Therefore, the perturbation of the water network together with direct and water‐mediated ligand‐protein interactions have to be considered in ligand optimizations to account for mode switching and to develop high activity ligands.^[51]^

## 2 Ligand Modifications Resulting in Functional Mode Switching

The development of allosteric ligands resulted in a breakthrough in mGlu modulation,[^4^, ^32^, ^33^, ^34^, ^35^, ^36^, ^53^, ^54^, ^55^] owing to numerous advantages such as improved subtype selectivity, better mimicking of physiological response, and larger freedom to operate (FtO) as compared to the optimization of glutamate analogues. However, controlled allosteric modulation proved to be challenging, as small modifications of compounds can result in a new receptor conformation and modification in the pharmacology, or complete loss of activity.[^4^, ^56^, ^57^]

The first reported functional switch was observed in the development of DFB, an mGlu_5_ ligand, for which the difluoro compound (3,3’‐difluorobenzaldazine) showed PAM activity, the dimethoxy analogue (DMeOB) showed NAM activity, and the dichloro cognate (DCB) was described as the first mGlu_5_ NAL^[58]^ (Table 1). These compounds bind in the so‐called allosteric MPEP binding site,^[58]^ which was reported to be the most common allosteric binding site in mGlu_5_. Interestingly, mode switching was observed almost exclusively in the case of ligands bound into this region. Rodriguez and co‐workers reported several mode‐switching compounds with acetylenic linker. During their exploration of SARs around the MPEP scaffold, they identified the second mGlu_5_ NAL, 5MPEP; however they also reported the first partial allosteric antagonists, M‐5MPEP and Br‐5MPEPγ, which only partially blocked the signaling despite occupying the allosteric binding site completely.^[59]^ Another partial antagonist (**1**) was found in a series with the 5‐(phenylethynyl)pyrimidine scaffold. 3‐Methyl substitution of the phenyl group of this scaffold resulted in NAMs (**2**, **4**); however, 4‐methyl substitution (**3**, **7**) and/or the introduction of an N‐methylamine substituent on the pyrimidine ring resulted in PAMs (**5**–**7**). The mode switching measured *in vitro* was also proved *in vivo*, as compound **5** showed efficacy in rodent models of schizophrenia.[^60^, ^61^] Several compounds were reported by the Vanderbilt University within the acetylene amide series, for which different substitution on the amide nitrogen resulted in PAMs (e. g., ML254, VU0361747[^62^, ^63^, ^64^]) PAs (e. g., VU0477573^[65]^) NALs (e. g., VU0478006 (ML353)^[66]^), and ago‐PAMs (VU0424465, (ML273);^[62]^ Table 1). Interesting observation was made during in vivo examination of VU0403602 where pan cytochrome P450‐mediated biotransformation of VU0403602 was discovered to produce a potent ago‐PAM mGlu_5_ ligand (M1).^[62]^ Furthermore, functional bias was also observed in this series: VU0477573 showed PA activity in the inhibition of glutamate EC_80_‐induced intracellular calcium release and full NAM activity when mGlu_5_‐mediated extracellular signal‐related kinases 1/2 phosphorylation was measured.^[65]^ A non‐acetylenic MPEP site PAM, ADX‐47273, was also described; however, this compound also showed intrinsic agonist activity, hence this was the first reported ago‐PAM.^[67]^ Changing the 4‐fluorophenyl moiety on this scaffold to 2‐pyridyl resulted in compound **8** as a pure PAM. Replacing the benzamide in **8** to cyclobutyl amide yielded NAM activity (**9**). Further optimization of **9** caused several kinds of mode switching depending on the ring size, on the simple substitution on the aryl ring (i. e., methyl, or fluoro), or the stereochemistry resulting in NAM (**11**, **15**, **19**), PAM (**10**, **14**, **18**), ago‐PAM (**13**, **17**), or partial antagonist (**12**, **16**) activity.[^67^, ^68^] Zhou and co‐workers described three HTS hits, *N*,*N’*‐(1,3‐phenylene)dibenzamide (**20**), 3‐(phthalimidyl)‐*N*‐(2‐hydroxyphenyl)benzamide (**25**) and *N*‐(3‐(1*H*‐benzo[*d*]imidazol‐2‐yl)‐4‐chlorophenyl)‐5‐bromofuran‐2‐carboxamide (**28**) as highly active mGlu_5_ NAMs.^[69]^ Beside NAMs (**21**, **22**, **29**), optimization of these scaffolds resulted in either PAMs with moderate activity (**23**, **24**, **30**) or partial antagonists (**26**, **27**). Compounds **25**–**27** showed structural similarity with CPPHA, a non‐MPEP site PAM, therefore the authors proposed that these compounds are the first reported CPPHA site NAMs. Nonetheless, these compounds showed MPEP displacement during further evaluation. Interestingly however, compound **30** was proposed to bind in a different binding site based on [^3^H]3‐methoxy‐5‐(2‐pyridinylethynyl)pyridine displacement data. One year later, this research group reported the first *in vivo* active PAM, VU0364289, which was developed from a potent mGlu_5_ NAM (**31**).^[70]^ Later, 4‐butoxy‐*N*‐(2‐fluorophenyl)benzamide (VU0040237) was disclosed as a non‐MPEP site PAM.^[10]^ The optimization of this HTS hit resulted in the first NAL (VU0365396), which binds in a different allosteric binding site.^[10]^ In the following years, several cases of mode switching were reported for the acetylenic chemotype. Modification of the 6‐(phenylethynyl)‐3,4‐dihydroisoquinolin‐1(2*H*)‐one scaffold (**32**) resulted in ago‐PAMs (**32**, **34**, **35**, **40**, **42**, **44**), pure PAMs (**33**, **36**, **38**, **41**, **43**, **45**, MRZ 3573), and partial antagonists (**37**, **39**).[^41^, ^71^] Moreover, a potent NAM was also reported (MRZ 8676).^[72]^ Later a new oxazolidinone‐based acetylenic chemotype was disclosed by Huang and co‐workers (**46**–**49**, BMS‐984923, BMS‐952048, BMS‐955829).^[73]^ Mode switching was also observed in the optimization of hit **46**; from the PAM activity of the initial HTS hit to ago‐PAM (**49**), NAM (**48**), or NAL (BMS‐984923). Mode switching was reported for bispyridine benzene derivatives, another compound set with acetylenic linker, where 1,3 substitution of the phenyl group resulted in NAMs (**50**, **52**); 1,4 substitution, however, resulted in partial antagonists (**51**, **53**).^[74]^

**Table 1:** mGlu receptor allosteric modulators where mode switching was observed for closely related structures.

Structure	Name	Substituent	Receptor subtype	Activity type (Figure 1)	Activity (IC_50_/ EC_50_)
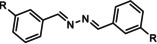	DMeOB DCB DFB	R=OMe R=Cl R=F	mGlu_5_	NAM NAL PAM	3.0 μm 7.6 μm 2.6 μm
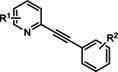	MPEP 5MPEP M–MPEP M‐5MPEP Br‐5MPEPγ	R^1^=6‐Me; R^2^=H R^1^=5‐Me; R^2^=H R^1^=2‐Me; R^2^=3‐OMe R^1^=5‐Me; R^2^=3‐OMe R^1^=5‐Me; R^2^=3‐Br	mGlu_5_	NAM NAL NAM PA PA	36 nm 201 nm 3.6 nm 134 nm 101 nm
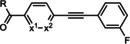	ML254 VU0477573 VU0424465 (ML273) VU0478006 (ML353) VU0403602 M1	X^1^=N X^2^=CH R= 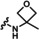 X^1^=N X^2^=CH R=N(Et)_2_ X^1^=N X^2^=CH R= 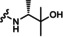 X^1^=N X^2^=CH R= 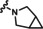 X^1^=N X^2^=CH R= 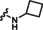 X^1^=N X^2^=CH R= 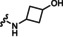	mGlu_5_	PAM PA ago‐PAM NAL PAM ago‐PAM	8.7 nm 32 nm 1.5 nm 18.2 nm 4 nm 400 nm
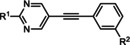	**1 2 3 4 5 6 7**	R^1^=H; R^2^=H R^1^=H; R^2^=3‐Me R^1^=H; R^2^=4‐Me R^1^=OEt; R^2^=3‐Me R^1^=NHMe; R^2^=H R^1^=NHMe; R^2^=3‐Me R^1^=NHMe; R^2^=4‐Me	mGlu_5_	PA NAM PAM NAM PAM PAM PAM	486 nm 7.5 nm 3.3 μm 21.1 nm 14.3 nm 21.1 nm 704 nm
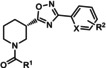	ADX47273 **8 9**	R^1^=4‐FPh; R^2^=4‐F; X=CH R^1^=4‐FPh; R^2^=H; X=N R^1^=*c*‐butyl; R^2^=H; X=N	mGlu_5_	ago‐PAM PAM NAM	170 nm 390 nm 8.7 μm
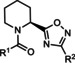	**10 11**	R^1^=*c*‐butyl; R_2_=3‐FPh R^1^=*c*‐butyl; R^2^=3‐ClPh	mGlu_5_	PAM NAM	700 nm 900 nm
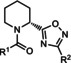	**12 13 14 15 16 17 18 19**	R^1^=*c*‐propyl; R^2^=3‐FPh R^1^=*c*‐propyl; R^2^=3‐MePh R^1^=*c*‐butyl; R^2^=3‐FPh R^1^=*c*‐butyl; R^2^=3‐MePh R^1^=*c*‐pentyl; R^2^=3‐MePh R^1^=*c*‐pentyl; R^2^=3‐FPh R^1^=*c*‐hexyl; R^2^=3‐FPh R^1^=*c*‐hexyl; R^2^=3‐MePh	mGlu_5_	PA ago‐PAM PAM NAM PA ago‐PAM PAM NAM	280 nm 80 nm 650 nm 370 nm 600 nm 310 nm 3.5 μm 2.5 μm
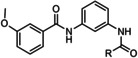	**20 21 22 23 24**	R=2‐ClPh R=2‐furanyl R=*c*‐propyl R=pyridine‐3‐yl R=pyridine‐4‐yl	mGlu_5_	NAM NAM NAM PAM PAM	2.62 μm 4.51 μm 2.23 μm 5.54 μm 1.87 μm
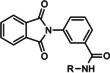	**25 26 27**	R=2‐hydroxyphenyl R=2‐pyridyl R=2‐fluorophenyl	mGlu_5_	NAM PA PA	420 nm 1.26 μm 1.05 μm
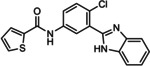	**28 29 30**	R^1^=5‐bromo‐2‐furanyl; R^2^=H R^1^=2‐hydroxyphenyl; R^2^=H R^1^=thiophene R^2^=Cl	mGlu_5_	NAM NAM PAM	85 nm 3.5 μm 2.2 μm
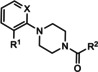	**31** VU0364289	R^1^=H; X=N; R^2^= 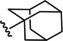 R^1^=CN; X=CH; R^2^= 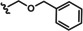	mGlu_5_	NAM PAM	540 nm 820 nm
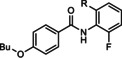	VU0040237 VU0365396	R=H R=F	mGlu_5_	PAM NAL	350 nm 100 nm
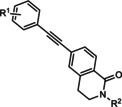	**32 33 34 35 36 37 38 39**	R^1^=H; R^2^=H R^1^=H; R^2^=Me R^1^=H; R^2^=*n*Pr R^1^=2‐F; R^2^=H R^1^=2‐F, 4‐F; R^2^=H R^1^=3‐F; R^2^=Me R^1^=3‐F; R^2^=*n*Bu R^1^=3F; R^2^= 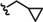	mGlu_5_	ago‐PAM PAM ago‐PAM ago‐PAM PAM PA PAM PA	50 nm 250 nm 160 nm 260 nm 850 nm 170 nm 54 nm 770 nm
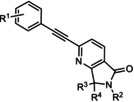	**40 41 42 43 44 45**	R^1^=H; R^2^=H; R^3^, R^4^==O R^1^=H; R^2^=pyridine‐2‐ylethyl; R^3^, R^4^==O R^1^=3‐F; R^2^=H; R^3^,R^4^==O R^1^=3‐F,4‐F; R^2^=H; R^3^,R^4^==O R^1^=H; R^2^=H; R^3^=R^4^=H R^1^=3‐F; R^2^=H; R^3^=R^4^=H	mGlu_5_	ago‐PAM PAM ago‐PAM PAM ago‐PAM PAM	5.9 nm 870 nm 35 nm 170 nm 51 nm 66 nm
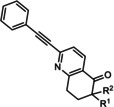	MRZ 3573 MRZ 8676	R^1^=H; R^2^=H R^1^=Me; R^2^=Me	mGlu_5_	PAM NAM	38 nm 23 nm
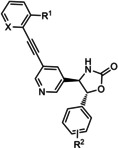	**46 47 48** BMS‐984923 **49** BMS‐952048 BMS‐955829	R^1^=H;X=CH; R^2^=H R^1^=H; X=CH; R^2^=2‐F R^1^=H; X=CH; R^2^=2‐F,6‐F R^1^=H; X=CH; R^2^=2‐Cl R^1^=H; X=CH; R^2^=4‐F R^1^=F; X=CH; R^2^=4‐F R^1^=H; X=CH; R^2^=2‐F,5‐F	mGlu_5_	PAM PAM NAM NAL ago‐PAM PAM PAM	0.7 nm 1.5 nm 27 nm 0.6 nm (Ki) 0.4 nm 10 nm 2.6 nm
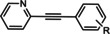	**50 51 52 53**	R=3‐ 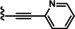 R=4‐ 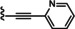 R=3‐ 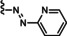 R=4‐ 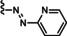	mGlu_5_	NAM PA NAM PA	430 nm 1.21 μm 960 nm 3.09 μm
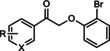	VU0219493 VU0448383	R=2‐OH,4‐OH; X=CH R=6‐Cl; X=N	mGlu_4_	PAM NAM	1.4 μm 8.2 μm

Interestingly, up to now, functional mode switching was reported almost exclusively in the case of mGlu_5_, except for two curious examples. The optimization of a potent mGlu_4_ PAM, VU0219493, resulted in VU0448383, the first mGlu_4_ NAM.^[75]^ Moreover, the modification of *N*‐(4‐fluorophenyl)‐7‐oxo‐7,7a‐dihydrocyclopropa[*b*]chromene‐1a(1*H*)‐carboxamide, an mGlu_2_ NAL (Table 2, compound **54**), produced mGlu_2_ NAMs^[76]^ (Table 2, compounds **55**, **56**).

**Table 2:** mGlu allosteric modulators for which subtype switching was observed for closely related structures.

Structure	Name	Substituent	Receptor subtype, activity type (Figure 1)	Activity (IC_50_/EC_50_)
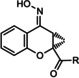	CPCCOEt (−PHCCC 54 55 56	R=OEt R=NH−Ph R=NH‐4‐F−Ph R=NH‐4‐Cl−Ph R=NH‐4‐Me−Ph	mGlu_1_ NAM mGlu_4_ PAM mGlu_2_ NAL/mGlu_3_ NAL mGlu_2_ NAM/mGlu_3_ PAM mGlu_2_ NAM/mGlu_3_ PAM	10.3 μm 3.1 μm 6.6 μm (*K*_i_) 0.8 μm/13.4 μm 1.5 μm/8.9 μm
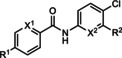	VU0001850 VU0361737	R^1^=OBu; R^2^=H; X^1^=CH; X^2^=N R^1^=H; R^2^=OMe, X^1^=N; X^2^=CH	mGlu_5_ PAM mGlu_4_ PAM	1.3 μm 240 nm
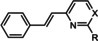	SIB 1893 TCN 238	X=CH; R=Me X=N; R=NH_2_	mGlu_5_ NAM mGlu_4_ PAM	370 nm 1 μm
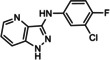	VU0418506	–	mGlu_4_ PAM	46 nm
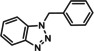	**57**	–	mGlu_2_ PAM	3 μm
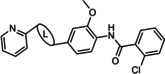	VU0415374 **58**	L=NH−CO L=N=N	mGlu_4_ PAM mGlu_5_ NAM	517 nm 8.6 nm
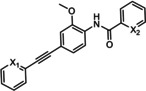	XGS‐RC‐009 **59**	X^1^=N; X^2^=C−Cl X^1^=C−Cl; X^2^=N	mGlu_5_ NAM mGlu_4_ PAM	24 nm 4.1 μm
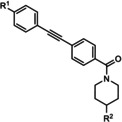	VU0092273 VU0463597/ ML289	R^1^=H; R^2^=OH R^1^=OMe; R^2^=CH_2_OH	mGlu_5_ PAM mGlu_3_ NAM	270 nm 1.5 μm

## 3 Structural Basis of Functional Mode Switching

The detailed understanding of the molecular mechanism of mGlu modulation is challenging owing to inconsistent SAR, mode and subtype switching, and biased modulation. However, available site‐directed mutagenesis results (e. g., in refs. [77–83]), the release of X‐ray structures for mGlu group I proteins’ 7TM region in complex with NAMs,[^51^, ^84^, ^85^, ^86^] and the cryo‐electron‐microscopic structure^[87]^ of the full‐length active like (potentiating antibody and agonist bound) and inactive mGlu_5_ significantly contributed to our understanding of the mechanism of allosteric modulation.

**Figure 2:**
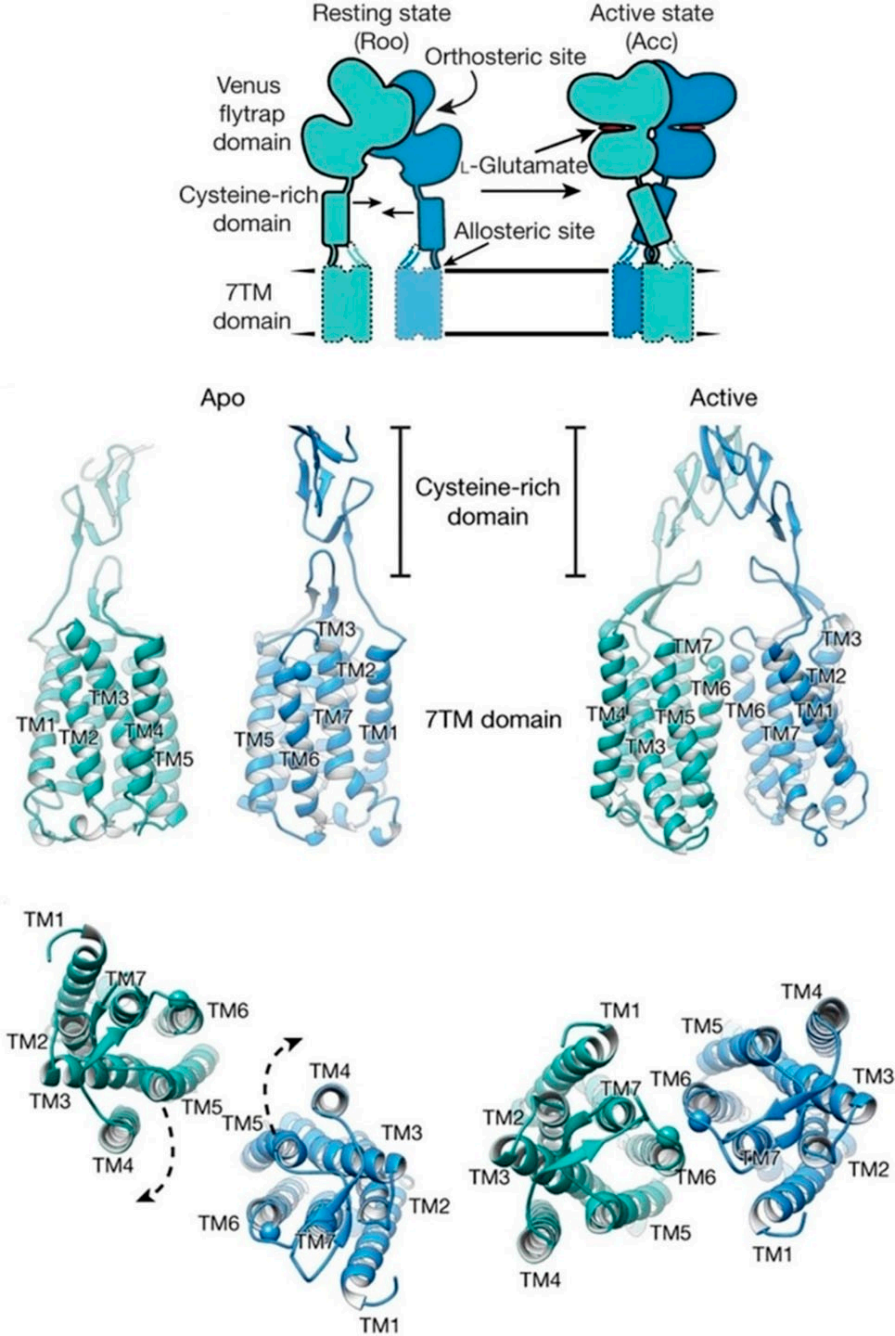
Top: Schematic view of mGlu_5_ activation with labeled domains. Middle: Side and bottom: top views of apo and active mGlu_5_ CRD and 7TM domains. Adapted with permission from ref. [87]. Copyright: 2019, Springer Nature.

Based on the available structural information, the homodimers of mGlus are crosslinked only through the Venus flytrap domain in the apo form, and activation results two major changes in this domain. The first is the closure of the two VFT lobes, and the second includes an inter‐subunit reorientation, which brings the cysteine rich domains (CRDs) to close proximity to each other.^[88]^ As the CRD is fairly rigid, this conformational change involves the approach of the two 7TM regions while they are rotated by 20°.^[87]^ This movement results in the establishment of a TM6–TM6 interface (Figure 2).

These observations are in line with the proposal that both inter‐ and intrasubunit rearrangements are required for full activation,^[89]^ however, they do not fully elucidate the connection among the formation of the interface, G protein coupling and signaling.

Before the first available X‐ray structure within the mGlu family the identification of key interactions of mGlu allosteric ligands with the 7TM of the protein in PAM and NAM complexes were addressed by site‐directed mutagenesis studies. These studies pointed out the presence of at least one common allosteric binding site between mGlu subtypes, because several residues were reported to be crucial for both positive and negative allosteric modulators in different subtypes. For example, positions 3.36a, 3.40c; 5.43a, 5.43c; 6.48a, 6.50c were essential in mGlu_1_, mGlu_2_, mGlu_4_ and mGlu_5_,[^78^, ^80^, ^81^, ^82^, ^90^, ^91^, ^92^, ^93^, ^94^, ^95^] residues 5.44a, 5.44c; 5.47a, 5.47c; 6.55a, 6.57c were important in mGlu_2_ and in group I mGlu receptors,[^78^, ^79^, ^80^, ^82^, ^91^, ^92^, ^93^, ^94^, ^96^, ^97^, ^98^, ^99^, ^100^, ^101^] 6.51a,6.53c was relevant in mGlu_4_ and group I receptors.[^78^, ^79^, ^80^, ^90^, ^91^, ^92^, ^95^] (GPCRdb generic residue numbering is used throughout the manuscript..[^102^, ^103^])

The impact of mutations on the effect of allosteric modulators were combined with affinity data obtained with radiolabeled allosteric modulators. Affinity and cooperativity determinants were mapped with the usage of the most well characterized representatives of allosteric modulator scaffolds in mGlu_1_ and mGlu_5_ structures.^[97]^ The most significant amino acids that modulated the affinity and cooperativity upon mutations in both mGlu_1_ and mGlu_5_ are: 5.43a, 5.43c; 5.44a, 5.44c; 6.48a, 6.50c; 6.51a, 6.53c; 6.55a, 6.57c, while residues at positions 3.36a, 3.40c; 3.40a, 3.44c; 5.47a, 5.47c; 6.52a, 6.54c; 6.55a, 6.57c; 7.45a, 7.40c; 7.46a, 7.41c are affecting only mGlu_5_ ligands, and mutation at position 7.38a, 7.33c changes the affinity and cooperativity only of mGlu_1_ ligands.[^78^, ^86^, ^96^] Interestingly mutations on several amino acids resulted in switch in allosteric modulator cooperativity (functional mode switch). Mutation at 6.48a, 6.50c results in cooperativity of NAMs of glutamate to positive,^[78]^ mutation at position 6.51a, 6.53c causes inverse cooperativity from the original both in the case of PAMs and NAMs.[^91^, ^92^] Moreover, point mutations at position 3.40a 3.44c; 6.44a, 6.46c; 7.45a; 7.40c also switch acetylenic PAMs to have neutral or negative cooperativity at mGlu_5_.[^78^, ^104^]

**Figure 3:**
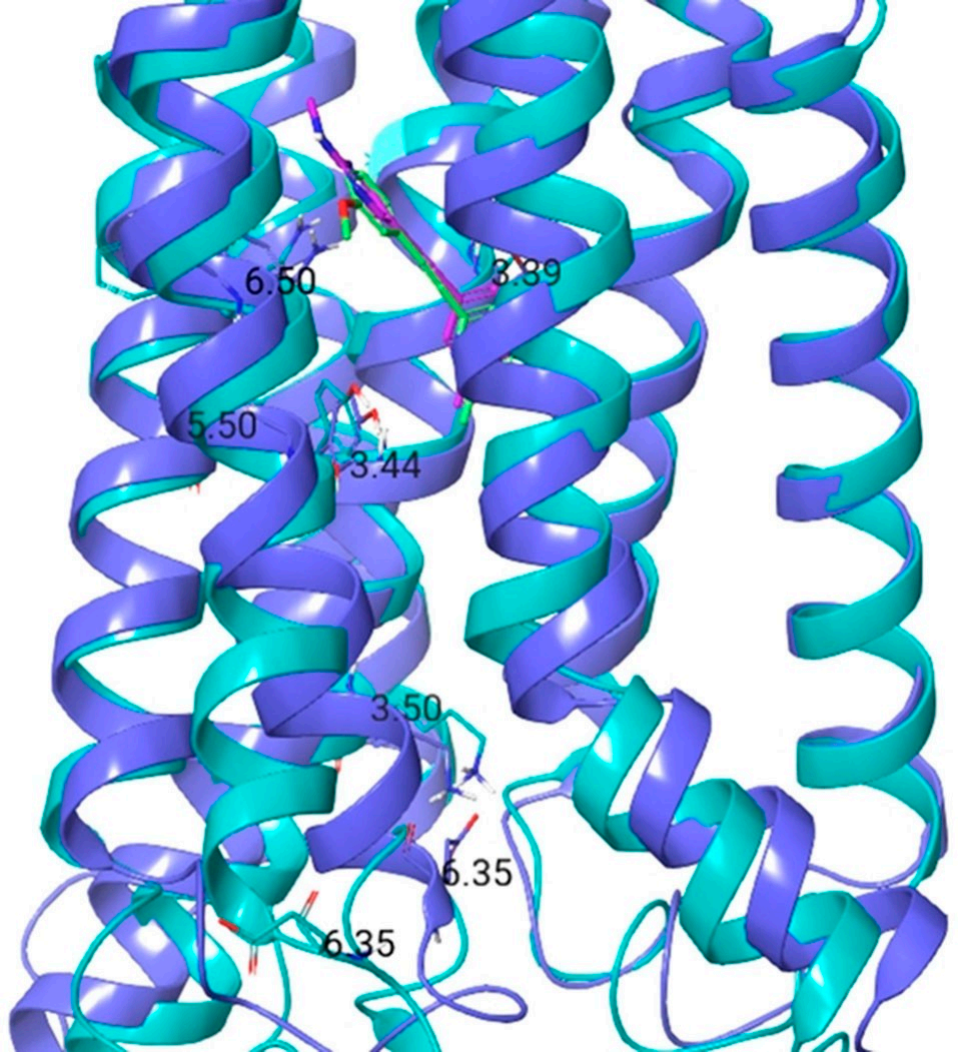
The 7TM regions of active and inactive mGlu_5_ with allosteric ligands **6** (magenta) and M‐MPEP (green), respectively. The active structure (the homology model of which was prepared based on the μ‐opioid receptor‐Gi protein complex (PDB ID: 6DDE^[112]^) as is written in ref. [52]) is depicted in cyan, the inactive (PDB ID: 6FFI) is in blue. Trigger switch, transmission switch, and “ionic lock” amino acids are represented as cyan and grey sticks.

The appearance of X‐ray structures of group I mGlus (4OO9,^[84]^ 5CGC, 5CGD,^[85]^ 6FFI, 6FFH,^[51]^ 4OR2^[86]^) opened the possibility to investigate the detailed mechanism of the 7TM intra‐subunit rearrangement needed for receptor activation. Since then, numerous structure based calculations have been applied.[^13^, ^47^, ^51^, ^52^, ^96^, ^105^, ^106^, ^107^, ^108^] These calculations were performed for mGlu proteins in complex with allosteric modulators. The most common and best‐characterized allosteric binding pocket within the mGlu family can be found in the 7TM region surrounded by the so‐called “trigger switch” (3.36a, 3.40c; 5.47a, 5.47c; 6.48a, 6.50c) and “transmission switch” (3.40a, 3.44c; 5.50a, 5.50c; 6.44a, 6.46c) amino acids, which were proposed to be crucial in the allosteric activation.[^105^, ^106^, ^109^, ^110^, ^111^] Molecular dynamics simulations showed that the 3.44c amino acid has a direct or water‐mediated interaction with 6.46c in the case of NAM and NAL binding.[^52^, ^105^, ^106^] This water molecule was observed in all available mGlu_5_ X‐ray structures and was found to have increased stability in complexes compared to the apo protein.[^51^, ^84^, ^85^] Nonetheless, these interactions cannot be formed in the PAM complexes owing to the bending of the TM6 in the active structure (Figure 3). 3.40c, a member of the trigger switch, was found to move toward TM6^[105]^ upon activation of mGlu_2_. In mGlu_5_, ionic interactions were observed among residue pair 3.50c–6.35c in the NAM structure, whereas it was not present in the PAM complex.^[108]^ The destabilization of the ionic lock was also observed in the mGlu_4_ PAM complex.^[111]^ Although these observations were reported for mGlu receptor monomers, they might be applicable to the dimers as well, because on the one hand, the computational results are in line with the site‐directed mutagenesis‐based experimental results detailed above, on the other hand, the active like cryo‐electron‐microscopic structure of mGlu_5_ shows that the establishment of TM6–TM6 interface only affects the top of the 7TM region far from the reported common allosteric binding pocket (Figure 2). The allosteric binding site of mGlus can be found in a functional water channel, and hence water is likely to play an important role in signal transduction. Therefore, calculations were also aimed at understanding the role of water molecules in ligand binding. These studies showed that most of the interactions between the ligand and the protein are water‐mediated, and hence the perturbation of the water network contributes to the observed ligand affinity and functional activity.[^106^, ^108^]

## 4 Ligand Modifications Resulting in Subtype Switching

The 7‐hydroxy‐iminocyclopropane‐chromene scaffold not only provided us with examples of functional switches, but a subtype switch was also observed among compounds of this series. The first reported 7‐hydroxy‐iminocyclopropane‐chromene analogue was CPCCOEt, which was an mGlu_1_ NAM.^[113]^ Later (−PHCCC^[114]^ phenylamide, and VU0359516,^[115]^ a pyridylamide analogue was described with mGlu_4_ PAM activity, then substitution of (−PHCCC on the phenyl ring resulted in mGlu_2_ NAM/mGlu_3_ PAM dual modulators^[76]^ (Table 2, compounds **55**, **56**, (−PHCCC). These latter ligands were the first allosteric modulators to display functionally opposite activities on the two group II mGlus. Subtype switches were also reported for several other ligands. VU0001850^[10]^ was described as an mGlu_5_ PAM and its close analogue, VU0361737,^[116]^ as an mGlu_4_ PAM. SIB1893^[117]^ appeared to be an mGlu_5_ NAM; however, TCN238^[118]^ turned to be an mGlu_4_ PAM. In addition, VU0418506^[119]^ and its close analogue 1‐benzyl‐1*H*‐1,2,3‐benzotriazole (**57**)^[120]^ were reported as optimized mGlu_4_ selective PAM and as a HTS hit for mGlu_2_, respectively. Pittolo and co‐workers described a potent mGlu_5_ NAM (**58**)^[121]^ during the development of photoisomerizable ligands from an mGlu_4_ PAM VU0415374.^[122]^ In the dark, 100 nM of **58** was able to antagonize the orthosteric activation of mGlu_5_; however, under continuous UV illumination, this compound was ten times weaker. This drop in the potency was reversible and subtype selective. A subtype switch was also described among acetylenic compounds. XGS‐RC‐009 and **59** were developed from VU0415374;^[122]^ however, they showed activity on different mGlus. XGS‐RC‐009 showed strong mGlu_5_ NAM activity, whereas **59** showed mGlu_4_ PAM activity.^[123]^ Another subtype switch was reported within this family: VU0463597/ML289, an mGlu_3_ NAM developed from VU0092273, a highly potent mGlu_5_ PAM.^[124]^ Interestingly, this compound showed the highest selectivity against mGlu_2_ (∼15‐fold).

## 5 The Impact of Allosteric Molecular Switches on Medicinal Chemistry Programs

The detection, validation and quantification of allosteric modulation is a permanent challenge in allosteric drug discovery. Binding and functional assays in various setups are used to explore the behavior of allosteric ligands.[^125^, ^126^] Binding assays are able to directly validate of allosteric mode of action and unmask the site of interaction; however, they are not able to provide information about efficacy modulation. Functional assays have the advantage of detecting a wider spectrum of allosteric behaviors including the modulation of affinity and efficacy. In addition, they are also useful to study probe dependence and saturation effects. The former describes the direction and degree of cooperativity between the allosteric modulator and the orthosteric ligand (probe), which might be, however, probe dependent.[^126^, ^127^, ^128^, ^129^] The latter expresses the limited influence of allosteric modulator caused by the cooperativity between orthosteric and allosteric sites. This also reduces the risk of over sensitization of the receptor by allosteric modulators.[^130^, ^131^]

Most commonly mGlu allosteric ligand detection relies on the determination of modulator concentration‐response curves with a single agonist concentration to acquire approximate modulator potency.[^43^, ^125^] Nevertheless, the potency of an allosteric modulator depends on the allosteric ligand affinity, cooperativity and intrinsic efficacy; moreover, it is influenced by the orthosteric agonist concentration.^[132]^ Therefore the determination of ligand affinity and cooperativity at target receptor and related subtypes during drug discovery is essential to achieve optimal selectivity.^[43]^ As many class C GPCRs lack selective radioligands, which obstructs the determination of ligand affinity by radioligand binding‐based methods, functional assays are used to evolve affinity and cooperativity estimates and to assess and optimize selectivity.[^125^, ^133^] Moreover, many studies use only a single orthosteric ligand for a single signaling pathway during ligand development, therefore only a limited part of the full pharmacology will be discovered. However, as it has come to the fore in recent years, allosteric ligands may have signaling‐pathway‐dependent effects. This phenomenon referred to as “biased modulation” and has been described for many GPCRs along with group I and group III mGlus.[^93^, ^97^, ^127^, ^134^, ^135^, ^136^, ^137^] Moreover, neutral allosteric ligands might be undetected owing to the neutral cooperativity with an orthosteric ligand, and in spite of their receptor affinity they may be categorized as inactive, as was demonstrated in the discovery of several neutral allosteric ligands for mGlu_5_.[^10^, ^59^, ^125^, ^137^] These data illustrate that the use of efficacy‐driven approaches and functional studies are inappropriate to describe allosteric modulator pharmacology and subtype selectivity completely, and that selectivity of allosteric modulators for class C GPCRs might be largely driven by cooperativity.

**Figure 4:**
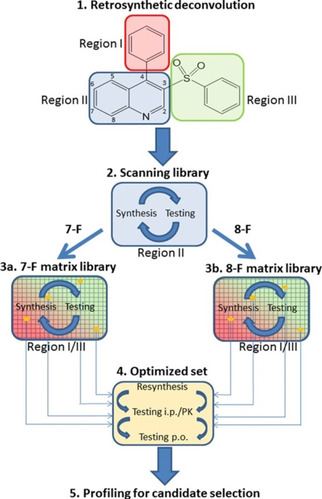
Implementation of the multiple parallel synthesis approach for the optimization mGluR5 NAMs. Retrosynthetic analysis (level 1) of the scaffold identified three regions (I, II and III) for further evaluation. The preferred substituents in region II (7‐fluoro and 8‐fluoro) were identified by the scanning library (level 2). Next, regions I and III were explored by matrix libraries (levels 3a and 3b). The most promising compounds identified from the matrix libraries were further characterized and optimized (level 4) to yield compounds profiled for candidate selection (level 5). Adapted with permission from ref. [139]. Copyright: 2017, American Chemical Society.

Mode switching affects the development and optimization of primary assays and the complexity of screening cascades, moreover, it influences the strategy of medicinal chemistry programs. Mode switching, together with the location and properties of the allosteric sites and the often steep or flat SAR make the optimization of GPCR allosteric modulators complex.^[43]^ As these effects are hardly predictable and the properties of allosteric sites are often challenged the ADME properties of the ligands, multidimensional parallel optimization strategies are typically considered.^[138]^ The implementation of this iterative, multidimensional parallel synthesis strategy has been recently exemplified by the optimization of an mGlu5 NAM to clinical candidate.^[139]^ The procedure starts with the retrosynthetic deconvolution of the starting point to identify regions to be optimized (Figure 4). Next, scanning libraries are used to explore the optimal set of substituents in each region. Having the optimized set of structural moieties identified, their combined effect is investigated by synthesizing and testing matrix libraries. Finally, most promising members of the matrix libraries are evaluated further and their head‐to‐head comparison provides candidates.

## 6 Summary

Allosteric modulation of mGlus has distinct advantages over orthosteric ligands in terms of subtype selectivity and reduced risk of receptor over sensitization, nonetheless, the optimization of these ligands proved to be challenging owing to often observed sharp and inconsistent SAR, functional selectivity, and molecular switches modulating the modes of pharmacology and subtype selectivity. Herein, we have reviewed allosteric molecular switches causing pharmacological mode and subtype switching, and summarized the available information on mGlu receptor‐activation mechanisms based on experimental and computational studies. It is emphasized that the allosteric binding site of mGlus might contain water molecules playing a significant role in the activation mechanism and mode switching that makes allosteric pharmacology poorly predictable. Recent developments in the structural biology of mGlus together with the availability of effective computational protocols might facilitate the discovery of novel allosteric ligands with designed pharmacology.

## Conflict of interest

The authors declare no conflict of interest.

## Biographical Information

*Zoltán Orgován received his BSc in chemistry from the Eötvös Loránd University of Budapest, Faculty of Science. He obtained his MSc degree in pharmaceutical engineering from Budapest University of Technology and Economics, Faculty of Chemical Technology and Biotechnology. He is currently pursuing his PhD at the Research Centre for Natural Sciences, Medicinal Chemistry Research Group under the supervision of Prof. György M. Keserű. His research interests include the development of covalent inhibitors and computational drug discovery*.

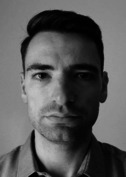

## Biographical Information

*György G. Ferenczy received his PhD in computational chemistry from the Eötvös University of Budapest. Following postdoctoral research at the Universities of Oxford (UK) and Nancy (France), he worked as a computational chemist and group leader first at Gedeon Richter and later at Sanofi. Since 2012, he has been a senior research fellow at Semmelweis University, and since 2013 at the Research Centre for Natural Sciences. His research interests include computational tools for extended biochemical systems and studying molecular interactions relevant to drug discovery. He has received the Overton and Meyer Award of the European Federation of Medicinal Chemistry*.

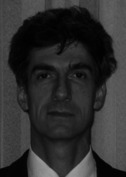

## Biographical Information

*György M. Keserű obtained his Ph.D. at Budapest. He worked for Sanofi before moving to Gedeon Richter. He contributed to the discovery of the antipsychotic Vraylar® (cariprazine), which is marketed in the US and EU. He served as director general of the Research Centre for Natural Sciences, Hungary and is now a full professor at the Budapest University of Technology and Economics. His research interests include medicinal chemistry and drug design. György received the Overton and Meyer Award of the European Federation of Medicinal Chemistry and was recently elected as a corresponding member of the Hungarian Academy of Sciences*.